# Maternal Expectations and Experiences of Labor Analgesia With Nitrous Oxide

**DOI:** 10.5812/ircmj.3470

**Published:** 2012-12-06

**Authors:** Hajar Pasha, Zahra Basirat, Mahmood Hajahmadi, Afsaneh Bakhtiari, Mahbobeh Faramarzi, Hajar Salmalian

**Affiliations:** 1 Fatemeh Zahra Fertility and Infertility Health Research Center, Babol University of Medical Sciences, Babol, IR Iran; 2 Community Medicine, Babol University of Medical Sciences, Babol, IR Iran; 3 Department of Midwifery, Babol University of Medical Sciences, Babol, IR Iran

**Keywords:** Labor Pain, Entonox, Pregnancy

## Abstract

**Background:**

Although there are various methods for painless delivery such as using entonox gas, most of the people are unfamiliar or concerned about it yet.

**Objectives:**

The purpose of this study was to assess maternal expectations and experience of labor analgesia with nitrous oxide.

**Patients and Methods:**

In a clinical trial study, 98 pregnant women in active phase of delivery were studied randomly in two groups (intervention group = 49, control group = 49) after obtaining written consent. Efficacy, experience satisfaction, and also expectation of pregnant women about entonox gas in two groups were compared, likewise in intervention group before and after using entonox gas.

**Results:**

Most of the pregnant women receiving entonox gas had less labor pain (91.8%), and were satisfied with it (98%). The severity of pain in the most of entonox user was moderate level (46.94%), while for the control group it was severe (55.10%) which was significant, 40.82% of the mother in entonox group had a severe pain and 10.20% had a very severe pain, whereas in the control group (55.10%) of the mother had a severe pain and 26.53% of the had very severe pain (P = 0.004). efficacy of labor pain was in moderate level in most cases. 49% of pregnant women receiving gas described their experience as a good and excellent. 80.9% indicated that they will request the mentioned painless method in the future. The amount of suffering from gas side effects was mild in most patients of intervention group (63%). Expectations of the majority of pregnant women in intervention group (before receiving gas) and control group for painless delivery were weak (65.3%, 40.9%). The percentage of positive expectations had increased after receiving entonox gas (P = 0.01). There was a difference between the expectations of intervention group receiving entonox gas and control group (P = 0.001). Positive expectations were more in intervention group than the control group. Most differences of expectations in intervention group before and after receiving the gas were about higher efficacy (P = 0.001), more satisfaction (P = 0.001), fewer complications (P = 0.001), information about gas as painless delivery method (P = 0.02), and also previous experience of intolerable labor pain (P = 0.04).

**Conclusions:**

This study has shown that using entonox gas caused less labor pain, favorable expectations and experiences and also more maternal satisfaction.

## 1. Background

Labor pain is one of the most acute pains that women experience during their lives (1, 2). Studies have shown that labor pain is the main reason for women to have the tendency for cesarean in spite of its higher complications. According to statistics declared by the Ministry of Health and Medical Education, the prevalence of cesarean in Iran is averagely 3 times more than global statistics. Nowadays, many attempts have been undertaken to reduce labor pain and there are various pharmaceutical and non-pharmaceutical methods for controlling labor pain. Painless normal delivery is gradually replacing cesarean in developed countries. This method,however, is less considered in Iran (3).One of the most common methods used for labor analgesia in most countries is a compound of inhaling nitrous oxide gas (50%), and oxygen(50%) as a safe and secure method with high efficacy, (4, 5) but lack of awareness about its advantages and disadvantages increased anxieties of expectant mothers about choosing this method, even they are rarely receptive; (2) While similar studies have shown no side effects for mother and fetus due to entonox gas use (6). It is used to any great extent in modern obstetric practice. The reasons for this include: the ease of administration of entonox gas, its lack of flammability, minimal toxicity, lack of effect on uterine contractility and safety for mother and fetal.

The women may experience the central nervous system effects of nitrous oxide like drowsiness, dizziness or lightheadedness (7).With regard to using entonox gas as one of the selected methods for diminishing labor pain and also reluctance of pregnant women to use this method, researchers were prompted to study the maternal expectations and experiences of labor analgesia with nitrous oxide, hoping that through the basic information gathered aboutentonox gas efficacy and expectations, experiences, and satisfaction of pregnant women about using the gas, its strengths and weaknesses could be discovered and used for educational programs.

## 2. Objectives

The purpose of this study was to assess maternal expectations and experiences of labor analgesia with nitrous oxide.

## 3. Patients and Methods

Our study is a clinical trial conducted on 98 pregnant women willing to participate in the study in the Maternity Ward in Shahid Yahyanejiad Hospital, Babol, in 2008-2009 after the approval of the Ethics Committee. The sample size of the study was based on similar researches, and according to statisticians, from 98 qualified samples, 49 were randomly selected for the intervention group (entonox gas recipients), and 49 pregnant women were categorized in the control group (without receiving gas). A written consent was obtained from all patients. The data gathering tool was a questionnaire including some information about demographic characteristics, severity of pain, efficacy, expectations, experiences and satisfaction of using the gas and also its related complications (headache, dizziness, blurred vision, drowsiness, light headedness, weakness, pricking sensation of tip of fingers and lips, mouth dryness, nausea and vomiting, etc.) which was completed by the researcher through an interview before and after the intervention. Expectation levels were divided into three groups: weak (< 50%), medium (50%-70%), and good(> 70%).The satisfaction rate and also the severity of labor pain control efficacy in intervention group after receiving the entonox gas were based on similar studies; Five-point Likert Scale criterion and qualitative options such as A:very good, B:good, C:medium, D:weak and E:none were in the questionnaire (8-12). A blend of inhaled nitrous oxide(N_2_O)50 percent and oxygen(O_2_) was used.

For sampling, at first, the necessary instructions were given to qualified women participating in the study about using the mask and how to breathe using the mask.As the active phase of laborstarted, which was defined as at least 4cms cervix dilatation, entonox gas was administered. Mothers started to inhale the gas at the beginning of the pain and they stopped when the pain ended; this continued until the start of the second stage of labor.

**Figure 1:**
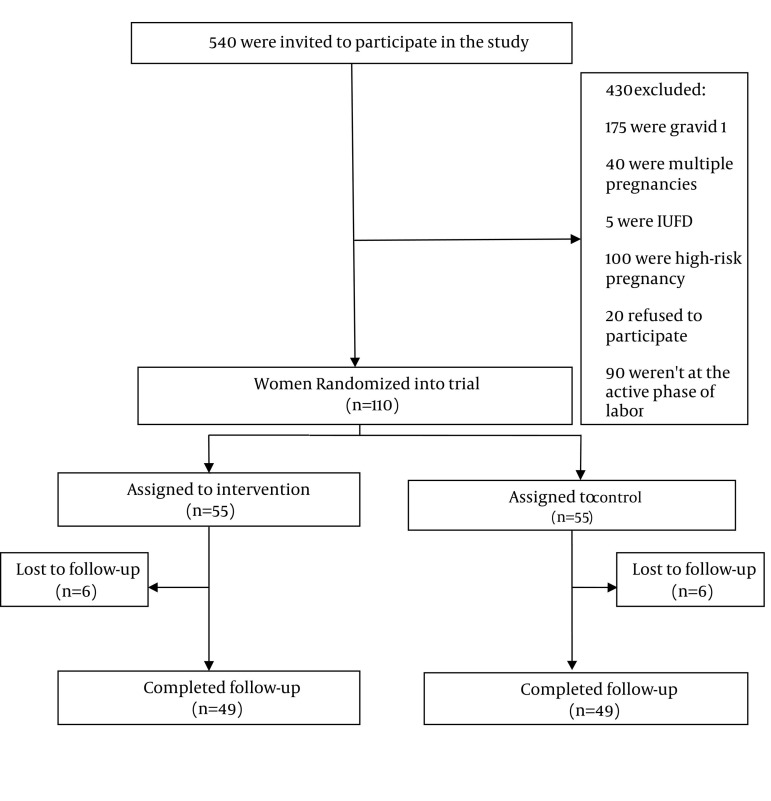
Flow Diagram of Participants Through Each Stage of Randomized, Controlled Trial

98 pregnant women with gestational age ranging from 37-42 weeks, being in the active phase of labor (with the minimum dilatation of 4 cm), pregnant for second time or more, were randomly enrolled in the study. Participants were selected among non-complicated term pregnancy, with a normal cephalic presentation. Women who were not able to keep their facial mask, and patients with known mental disease and high risk pregnancy(multiple pregnancy, placenta and fetus problems, internal disease or surgery in mother, fetal distress, clear stenosis of pelvic diameters, were excluded from the study. Figure 1 shows the flow diagram of participants through each stage of randomized, controlled trial.

Finally, 98 participants remained until the end of the study (49 intervention group, 49 control groups). After getting the information, the data was analyzed through descriptive-analytical statistic by SPSS software.Demographic variables (i.e., age, job, educational level, financing status, gravidity) were summarized to characterize the study population. Statistical analyses were performed using t-test (i.e., mean of age), Chi Square (i.e., other demographic variables, severity of labor pain, expectations about gas between groups), and ANOVA (mean expectations in intervention group before and after using the gas, and efficacy, satisfaction ,complication severity rate, tolerable labor pain, and awareness about entonox gas analgesics), Mc Nemar test (expectations about gas before and after use in intervention group) to determine probable significant associations, and a P value less than 0.05 was considered significant.

## 4. Results

The majority of the pregnant women in both groups were between 20-34 years of age. The mean ages of the patients in intervention and control groups were 27.96 ± 5.08, 27.9 ± 4.46respectively.Most of the subjects were experiencing second pregnancy and after that primary Para, housewivesand after that with diploma or higher education, and average financial status. Almost half of the pregnant women had a previous experience of intolerable labor pain. The majority of pregnant women only knew that entonox gas was one of the methods used for reducing labor pain. The mostimportant sources of getting information about entonox gas were midwives. Most pregnant women preferred specialists and then midwives for supervising the process of getting gas (Table 1).

**Table 1:** The Frequency Distribution of Demographic Characteristics of the Pregnant Women in the Intervention Group (Receiving Gas) and the Control Group (Without Receiving Gas)

Characteristics	Intervention Group, No. (%)	Control Group, No. (%)	Total Number No. (%)	P value
**Age**				
< 20	3 (6.1)	1 (2)	4 (4.1)	P = 0.247
20-34	40 (81.6)	45 (91.9)	85 (86.7)	
> 34	6 (12.3)	3 (6.1)	9 (9.2)	
**Job**				
Housewife	40 (81.6)	45 (91.8)	85 (86.7)	P = 0.099
Employed	9 (18.4)	4 (8.2)	13 (13.3)	
**Level of Education**				
Illiterate and LowLiteracy	3 (6.1)	17 (34.8)	20 (20.4)	P = 0.002
Junior and Senior High School	18 (36.7)	16 (32.6)	34 (34.7)	
Diploma and University	28 (57.3)	16 (32.6)	44 (44.9)	
**Financing Status**				
Low	9 (18.3)	17 (34.8)	26 (26.5)	P = 0.105
Moderate	29 (59.2)	27 (55.1)	56 (57.2)	
High	11 (22.4)	5 (10.1)	16 (16.3)	
**The Previous Experience of Labor Pain**				
Intolerable	24 (49)	19 (38.8)	43 (43.9)	P = 0.243
Tolerable	25 (51)	30 (61.2)	55 (56.1)	
**Informed about the analgesic effects of Nitrous Oxide gas**				
Yes	39 (79.4)	31 (63.3)	70 (70.2)	P = 0.125
No	10 (20.4)	18 (36.7)	28 (29.8)	
**The source of Receiving Information**				
Physician	3 (6.1)	3 (6.1)	6 (6.1)	P = 0.685
Midwife	26 (53.1)	24 (48.9)	35 (35.7)	
Others	20 (40.8)	22 (45)	57 (58.2)	
**Gravidity**				
2	38 (77.6)	30 (61.2)	68 (69.4)	P = 0.251
3	8 (16.3)	14 (28.6)	22 (22.4)	** **
≥ 4	3 (6.1)	5 (10.2)	8 (8.2)	

Level of labor pain before intervention was statistically equal between groups; however, it showed difference after intervention. Pain severity was lower in patients who received nitrous oxide (P = 0.004).The severity of pain in the most of entonox users in active phase of labor was moderate level (46.94%), while being severe for the control group (55.10%); 40.82% of the mothers in entonox group had a severe pain and 10.20% had a very severe pain, whereas in the control group 55.10% of the mothers experience a severe pain and 26.53% of whom had very severe pain. The majority of pregnant women using entonox gas received efficacy of entonox gas in a different level and had less labor pain, and only 2% of them were unsatisfied (Table 2).

**Table 2:** The Frequency Distribution of the Efficacy and Satisfaction Rate Using of Nitrous Oxide Gas on Labor Pain

Scale	Efficacy No. (%)	Efficacy No. (%)
**Excellent**	6 (12.2)	7 (14.3)
**Good**	5 (10.2)	17 (34.7)
**Medium**	21 (42.9)	14 (28.6)
**Low**	12 (24.5)	10 (20.4)
**No Relief/No**	5 (10 .2)	1 (2)

**Figure 2:**
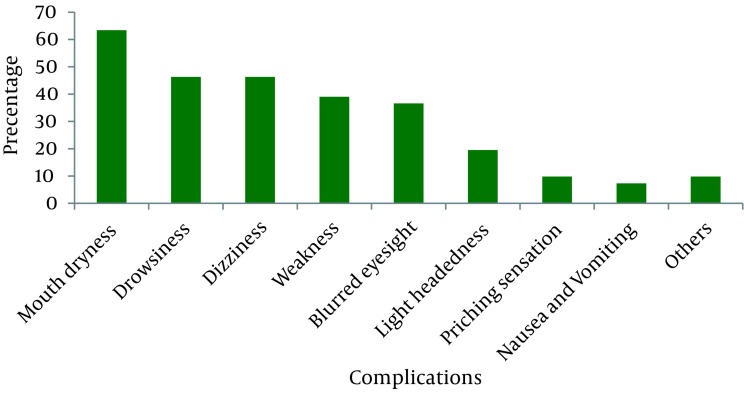
The Frequency Distribution of Intervention Group (After Receiving the Gas) and Control Groups Based on the Type of the Complications of the Nitrous Oxide

80.9 percent mentioned that they will ask for the analgesic method in the future. 93.8% of the women receiving the gas experienced some complications with different degree caused by the use of the entonox gas (Figure 2).

The irritation severity of the above mentioned complications was slight in most cases (63%) and it was severe only in a very small fraction (8.7%). Expectation levels of pregnant women were weak in 40.9% of cases, medium in 17.4%, and good in 28.5%. Generally, the level of expectation was low in intervention (before receiving the gas) and control groups. There was significant statistical difference between the expectations of women within and between groups (Table 3).

**Table 3:** The Frequency Distribution of the Expectations About Nitrous Oxide Gas Withinand Between Groups (P = 0.01, P = 0.001)

Expectations	Intervention Group (Before Receiving Gas), No. (%)	Intervention Group (After Receiving Gas), No. (%)	Control Group, No. (%)
**Weak**	20 (40.9)	8 (16.3)	32 (65.3)
**Medium**	11 (22.4)	6 (12.2)	7 (14.3)
**Good**	18 (36.7)	35 (71.5)	10 (20.4)

23.5% of pregnant women predicted that receiving entonox gas is dangerous for mother, and fetus (22.4%) . 12.3% of them stated that the gas would cause labor disorders, 11.2% said that it would prolong the labor and 9.2% believed that it increases in cesarean. Positive expectations increased after receiving the gas (Table 3), and the rate of mothers’ positive expectations about the entonox gas such as its safety, its dominance on pain, stress and pain decrease, the tendency to use the gas in next pregnancies, suggesting it to others, significantly increased and negative expectations such as high risk for mother, fetal, neonate and labor disorders decreased after receiving gas. Also, there was difference between the expectations of women in intervention group (before and after using the gas), and the efficacy of entonox gas (P = 0.001), satisfaction of its use (P = 0.001), its complication severity rate (P=0.001), the previous experience of an intolerable labor pain (P = 0.04), and awareness aboutentonox gas analgesics (P = 0.02).

## 5. Discussion

The findings of this study showed that the severity of labor pain in two groups was different and entonox gas had appropriate efficacy on labor pains. The severity of pain in most of entonox users was moderate level, while being severe for the control group and it was statistically significant. Moreover, those who experienced high level of entonox gas diminishing effects on labor pain, had higher positive expectations. There was a significant statistical relation between pregnant women’s expectations and diminishing effects of entonox gas on labor pain .In a similar study, pain severity was lower in patient who received nitrous oxide, (13) and a considerable pain relief was reported due to entonox gas use (7). The rate of pain relief was between 40-92 percent after receiving the gas (7, 14),and almost one third of the women's attitude changed about theentonox gas after receiving it (7). It seems that a serious endeavor should be undertaken to use the suitable different methods on diminishing labor pain.

The studies have shown that the majority of pregnant women receiving the entonox gas were satisfied with its analgesic effects and reported good experiences about it. There was a significant statistical relationship between intervention group expectations after using the gas and their level of satisfactory .Those that had good expectations about receiving the gas had the highest level of satisfaction in intervention group. Another similar study showed a high level of satisfaction of entonox gas users with its labor analgesic effects (1, 11, 15, 16). Basically, good analgesics can be used as a prognostic factor for satisfaction (17).

This study showed that the majority of pregnant women receiving entonox gas had some slight complications caused by the gas. The most common complication of gas was mouth dryness. Another study reported that the safety of entonox gas was confirmed on vast clinical levels and its complications were very few which included mouth dryness (70%), headache (25%), dizziness (25%), drowsiness (34.9%), nausea (9.6%), and drowsiness and mouth dryness (6, 18-20). In fact, the entonox gas is an important, reliable and prevalent choice for childbirth analgesia and should be more available for pregnant women (21, 22). Also, there was a significant statistical relationship between the expectations of intervention group before and after receiving the gas and its severity of irritation; thus, those women who had less irritation caused by the gas were more expected. This necessitates the consideration of the side effects of gas like mouth dryness and ways of treating these complications like mouth dryness. The study showed that midwives were the main source of getting the needed information about the entonox gas in intervention group with higher percentage in comparison with the control group.

Also, the majority of pregnant women preferred specialists to midwives for supervising the process of receiving the entonox gas. Asgarzadeh mentioned in his study that 53% of pregnant women chose the specialists and 29.3% of them selected midwives for labor analgesia. Since natural childbirth is an indisputable right of any woman and relieving the pain is the duty of any specialist or midwife, it is necessary for the personnel of medical centers especially midwives and specialists to try harder to perform painless childbirth, so the control of labor pain should become the first and the most important responsibility of health care team (23).

The findings of this study had shown that a high percentage of pregnant women in intervention group (before receiving the gas), and control group had weak expectation about labor analgesia with the entonox gas. Foroud and colleagues reported that although many endeavors have been done to undertake various labor analgesic methods in medical centers of our country, there are some wrong imaginations about the nature and the ways of reducing the labor pain and unfortunately most of the people are worried about it (3). Basically, labor pain and the methods to relieve it are one of the main anxieties of pregnant women and their families (24), which naturally influences individuals conceptions and expectations. Obviously, the role of awareness to improve the behavioral models is very important and it is the first stage in choosing positive attitude and getting a healthy behavior (25).

The results of the present research showed a significant statistical difference between expectations of the pregnant women in intervention and control groups as the positive expectations of women in intervention group was more than the control group. Also, the demographic characteristics of the control group such as profession, education, and financial status were different from intervention group with a higher percentage. Other similar studies have shown that the demographic characteristics such as education level, financial and social status can have effect on awareness, attitude, and function, even on views of people (26).

The data have shown a significant difference in expectationsof the intervention group before and after receiving the entonox gas. Positive expectations increased in intervention group after receiving the gas. Mothers’ positive expectations such as safety of the gas, dominance on pain, stress relief, pain relief, and tendency for receiving the gas for subsequent pregnancies and advising it to others, considerably increased. In a study carried out in Netherlands, it was observed that the majority of pregnant women had positive opinion about pharmaceutical pain relief after receiving the gas (27). The collected data showed that, there was a significant statistical difference between the expectations of intervention group before and after receiving the gas.with knowing that, the entonox gas was one of the methods of labor analgesia, so the highest mean difference of expectations in intervention group before and after receiving the gas was related to those unaware of entonox gas analgesia. The investigations have shown that lack of information was one of the reasons of this negative attitude (28). Patients were fearfulness and anxious about using the pharmaceutical methods due to their ignorance, and their attitude towards painless labor was associated with fear and apprehension (29, 30).

The investigations have shown that half of the women had experienced an intolerable pain on their previous delivery. The percentage of the previous intolerable labor pain in intervention group was higher than the control group. In addition to that, there was a significant statistical relationship between the expectations of the intervention group before and after receiving the gas, and the experience of previous labor pain. Most of the women in intervention group with the experience of previous intolerable labor pain, had good expectations and predictions.

Basically, the severity and difficulty of labor pain is obvious to everyone. Women experiencing delivery said that it was extremely painful (25). Similar studies showed that 58.7% of the women experienced intolerable labor pain and 41.3% of them said it was tolerable (29). Also, experience of the previous labor pain and fear of difficult delivery were the reasons for choosing the cesarean and avoidance of vaginal delivery (30). It seems that in this research, the experience of previous unpleasant labor pain and reduced labor pain in present delivery has increased the positive expectations about using the gas.

Regarding the suitable efficacy and few complications of the extonox gas on labor pains, pleasant maternal expectations and experiences, and also the increase in positive expectations after receiving the gas, women's subsequent pleasant experience and boosted positive expectations and attitudes, necessitates a proper planning and establishing a consultation system to provide information about various methods of labor analgesics and their functions especially the entonox gas, to hopefully increase the tendency of pregnant women to undergo natural delivery without bearing severe labor pains.
